# Metastatic Oncocytic Renal Neoplasm Presenting as Gastrointestinal Bleeding

**DOI:** 10.7759/cureus.83797

**Published:** 2025-05-09

**Authors:** Hasan Sqour, Abdul-Rahim Shilbayeh, Yasmin Gerais, Abdulrahman Sqour, Munther Hammad, Abdul-Rahman I Abusalim, Dolly Ewili, Marwah Alchalabi, Mohammad Salameh, Abdelfattah Saleh, Sammy Hamad, Laura Hamad, Fares Hamad, Bachar Hamad

**Affiliations:** 1 Internal Medicine, Ascension Saint Joseph Hospital, Chicago, USA; 2 Internal Medicine, John H. Stroger, Jr. Hospital of Cook County, Chicago, USA; 3 Gastroenterology, Saint Joseph Medical Center, Joliet, USA; 4 Internal Medicine, Yarmouk University, Irbid, JOR; 5 Internal Medicine, MedStar Washington Hospital Center, Washington, D.C., USA; 6 Internal Medicine, University of Wisconsin School of Medicine and Public Health, Madison, USA; 7 Internal Medicine, Hamilton Medical Center, Dalton, USA; 8 Internal Medicine, Islamic Hospital, Amman, JOR

**Keywords:** colon metastasis, gastrointestinal bleeding, gi bleed, immunotherapy, metastatic renal cancer, oncocytic neoplasm, renal oncocytoma, sigmoid colon

## Abstract

Renal cancer (RC) is known for its diverse clinical presentations and unpredictable behavior. While it often metastasizes to common sites such as the lymph nodes, lungs, bones, and liver, its potential to metastasize to the gastrointestinal tract (GIT) is relatively rare. We report an unusual case of an 87-year-old male patient with a history of metastatic oncocytic renal neoplasm who presented with intermittent rectal bleeding. Colonoscopy revealed a bleeding mass in the sigmoid colon. Biopsy confirmed metastatic renal neoplasm, consistent with prior pathology. Immunohistochemistry was positive for AE1/AE3, CAIX, and PAX-8, supporting renal origin. Recent CT imaging showed enlargement of the left renal mass with direct extension into the descending colon and associated lymphadenopathy. Metastasis of RC in the GIT, especially the colon, is rare and even more so in oncocytic subtypes. This case underscores the importance of considering RC in patients with GI bleeding and a history of renal neoplasm. Early recognition and individualized, multidisciplinary management are crucial for optimal outcomes.

## Introduction

Renal cancer (RC) is known for its diverse clinical presentations and unpredictable behavior. While it often metastasizes to common sites such as the lymph nodes, lungs, bones, and liver, its potential to metastasize to the gastrointestinal tract (GIT) is relatively rare [1-3]. When RC does involve the GIT, it can manifest as gastrointestinal bleeding, an uncommon but significant clinical scenario [2]. Renal oncocytoma is a benign epithelial neoplasm of the kidney that comprises 3-8% of all renal neoplasms [4]. The incidence of renal oncocytoma is relatively low, with an age-standardized incidence of 0.3 per 100,000 per year [5]. Although renal oncocytomas have been associated with an excellent prognosis given their benign nature, these tumors do have malignant potential and have been reported to metastasize [4]. We present a case describing an unusual occurrence of metastatic oncocytic renal neoplasm presenting as rectal bleeding due to a mass in the descending colon.

## Case presentation

An 87-year-old male with a history of metastatic left-sided oncocytic renal neoplasm, previously treated with cryoablation and pembrolizumab, presented for an outpatient colonoscopy to evaluate rectal bleeding. The patient reported intermittent hematochezia over the preceding weeks. On physical examination, there were no acute findings. Laboratory tests revealed a hemoglobin level of 9.5 g/dL, consistent with the patient's baseline.

**Figure 1:**
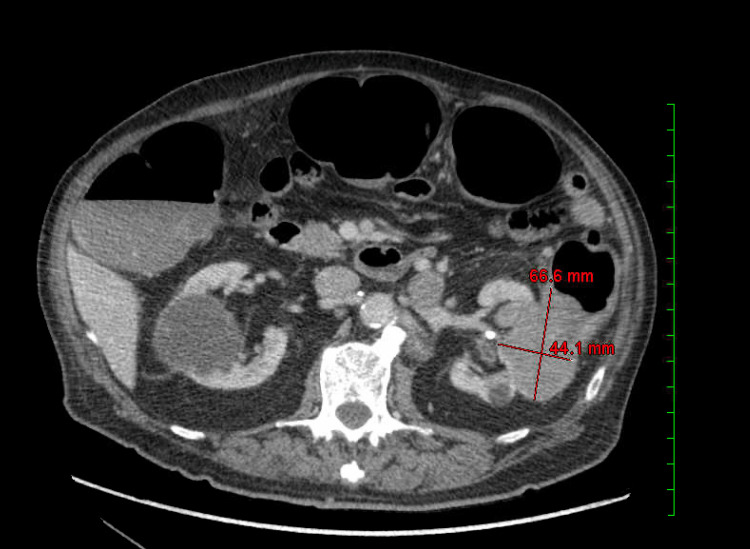
Abdominal CT scan The abdominal CT scan found that the patient's known left renal mass is showing further enlargement, now measuring up to 6.6 cm and extending into the adjacent descending colon.

**Figure 2:**
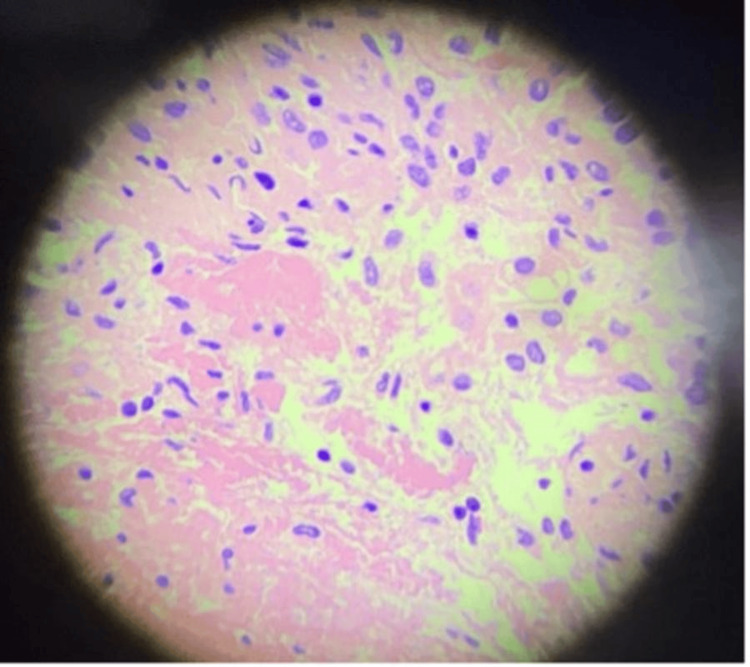
Histopathology Histopathology shows nests of oncocytic cells with granular eosinophilic cytoplasm and prominent nucleoli, consistent with metastatic oncocytic renal neoplasm.

**Figure 3:**
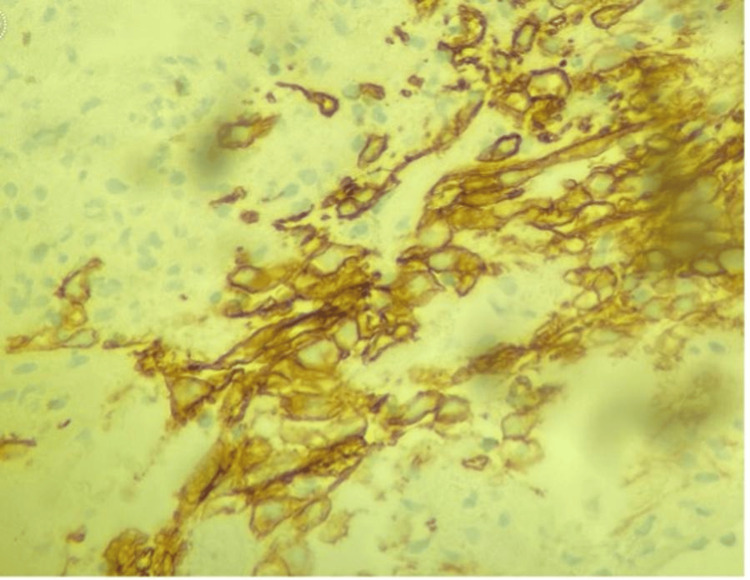
Immunohistochemistry Immunohistochemical staining was positive for AE1/AE3, CAIX, and PAX-8. Negative for p40.

Recent computed tomography (CT) imaging of the abdomen demonstrated progression of the left renal mass, now measuring up to 6.6 cm and extending into the adjacent descending colon, accompanied by abdominopelvic lymphadenopathy (Figure 1). These findings raised suspicion of disease progression. Colonoscopy revealed a mass within the sigmoid colon with associated bleeding. Biopsy of the mass confirmed metastatic cancer consistent with the histopathology of the patient's known RC (Figure 2). Immunohistochemical staining was positive for AE1/AE3, CAIX, and PAX-8, while negative for p40 (Figure 3), supporting the diagnosis of metastatic RC.

Given this new finding of disease progression and metastasis, a multidisciplinary team involving urology, colorectal surgery, gastroenterology, and oncology was arranged. All treatment options, including surgical resection, targeted therapies, and immunotherapy, were discussed with the patient. After careful consideration, the patient preferred to continue immunotherapy with pembrolizumab as an outpatient and refused surgical options.

## Discussion

Renal cell carcinoma is the most common type of RC, typically affecting individuals aged 60 to 70, with a higher incidence in men (the ratio is approximately 3.2) [6]. Renal cell carcinoma subtypes are classified based on histology and include clear cell (the most common subtype, 75-80%), papillary (10%-15%), and chromophobe (5%) [7]. Other rare histological subtypes of renal cell carcinoma include collecting duct, medullary, mucinous tubular and spindle cell, and Xp11.2 translocation/TFE3 [8-10].

Renal oncocytoma is often referred to as a benign epithelial neoplasm of the kidney that comprises 3-8% of all renal neoplasms [4]. Although renal oncocytomas have been associated with an excellent prognosis given their benign nature, these tumors do have malignant potential and have been reported to metastasize [4]. Local invasiveness, including perinephric fat and renal vein involvement, has been described with renal oncocytomas [4]. However, this reportedly did not alter the favorable prognosis of those patients. Distant metastasis has been reported to involve the liver and bone [4]. This suggests that despite being classified as a "benign kidney tumor," renal oncocytomas do have a low potential for malignancy.

It is important to mention that renal oncocytoma and chromophobe renal cell carcinoma are both oncocytic renal tumors that can appear similar on histological and radiological evaluation; however, they differ markedly in behavior and prognosis [4,5]. Differentiating between them can be challenging but is achievable through histological, immunohistochemical, and molecular analyses. Although a detailed discussion of these diagnostic methods is beyond the scope of this report, immunohistochemical and molecular features are particularly useful. For example, chromophobe renal cell carcinoma typically expresses cytokeratin 7 and exhibits characteristic chromosomal losses, whereas renal oncocytomas generally lack these markers [10]. Both tumors may express CD117, but S100A1 tends to be positive in renal oncocytoma and negative in chromophobe renal cell carcinoma [11,12]. Therefore, accurate diagnosis using a combination of pathological and molecular assessments is crucial to prevent overtreatment of benign oncocytomas and to ensure appropriate management of potentially malignant chromophobe renal cell carcinoma. However, despite the extensive analysis techniques available, some cases cannot be definitively differentiated even with extensive histologic, immunohistochemical, and molecular studies. In such cases, a descriptive diagnosis of "oncocytic renal neoplasm" is often used to reflect the uncertainty, which was the pathological diagnosis in our case [13].

RC can metastasize through lymphatic, hematogenous, transcoelomic, or direct invasion pathways [14]. While common sites of metastasis include the lungs, lymph nodes, bones, and liver, metastasis to the GIT is relatively rare, with the colon being an even less common site compared to the stomach and small bowel [2,3]. Of all RCs, the clear cell subtype metastasizes the most frequently to the GIT [15]. A noteworthy part of our case is that it involves an oncocytic renal neoplasm that directly invaded the colon, which is particularly rare.

Our case is also notable for an atypical presentation of an oncocytic renal neoplasm presenting as a lower gastrointestinal bleed. Clinical presentation of RC often involves a combination of flank pain, abdominal mass, and hematuria, although this complete classic triad is often seen in only about 10% of cases [16]. With the widespread use of advanced radiological imaging, RC is currently being diagnosed incidentally in 37-61% of cases [1,17-19]. Gastrointestinal bleeding is a rare presentation of RC; it is also rarely a presentation of progression/metastasis of RC [14,15].

Management of RC involving the GIT requires a multidisciplinary approach. Treatment options include surgical resection, targeted therapies, and immunotherapy [8,20,21]. Goals of treatment are to alleviate symptoms, control disease progression, and prolong survival. In this case, the patient opted to continue outpatient immunotherapy with pembrolizumab and declined further surgical intervention. This decision highlights the importance of individualized treatment planning, considering patient preferences and overall health status.

## Conclusions

This case highlights a rare presentation of metastatic oncocytic renal neoplasm to the descending colon, manifesting as gastrointestinal bleeding. Clinicians should consider RC as a potential source of metastatic disease in patients with known RC and unusual gastrointestinal symptoms. Early recognition and diagnosis are crucial for optimizing management strategies and improving patient outcomes.
